# Trends and patterns of deaths, injuries and intentional disabilities within the Libyan armed conflict: 2012-2017

**DOI:** 10.1371/journal.pone.0216061

**Published:** 2019-05-10

**Authors:** Mohamed A. Daw, Abdallah H. El-Bouzedi, Aghnyia A. Dau

**Affiliations:** 1 Department of Medical Microbiology & Immunology, Faculty of Medicine, University of Tripoli, Tripoli, Libya; 2 Department of Laboratory Medicine, Faculty of Biotechnology, Tripoli University, Tripoli, Libya; 3 Department of Surgery, Tripoli Medical Centre, Faculty of Medicine, University of Tripoli, Tripoli, Libya; Bielefeld University, GERMANY

## Abstract

**Background:**

The consequences of armed conflicts impose considerable burdens on the economy and health care services, particularly in countries that are not equipped to deal with them, such as in the Middle-East, and North African countries. Little is known about the burden of mortality and injury resulting from the Libyan armed conflict. This study aimed to determine the trends and patterns of mortality, injury and disabilities directly associated with the Libyan armed conflict and analyze the geographic variation within the country during 2012–2107.

**Methods:**

Data on conflict-related deaths, injuries, and disabilities were obtained from the national registry offices. The information included date, place, and demographic information. A questionnaire was also used to obtain information from the affected individuals and their families. National and regional trends of mortality, injury and disabilities were calculated. Spatial analysis was performed using geographic data available on all documented cases to analyze clustering of mortality and injury.

**Results:**

A total of 16,126 deaths and 42,633 injuries were recorded with complete information during the Libyan conflict from 2012 till 2017. The overall mortality rate was 2.7/1000 population and injury rate was 7.1/1000. The overall male-to-female ratio of mortality and injury was 4.4:1; 42.3% were single and aged 20–30 years old, and 26.4% were aged 31–40 years. Moreover, injuries resulted in death in 20.1% of cases and disability in 33.5% of the cases. Most of the disabilities were caused by blasts, while gun shots resulted in more deaths. The overall mortality and injury rates were highest during 2015–2017. These rates were highest in the eastern region. Injuries were most concentrated in Benghazi and Derna in the east, followed by Sert and Musrata in the central region.

**Conclusions:**

Conflict-related mortality, injury and disability has inflicted a heavy burden on the Libyan society that may persist for a long time. The rates of these casualties varied in time and place. National, well-planned efforts are needed to address this serious situation and its consequences.

## Introduction

Many parts of the world are involved in armed conflicts, the most prominent of which are in Arab countries, including Syria, Iraq, Yemen and Libya. Armed conflicts always impose heavy burdens of death, injury and disability [1]. Globally, it has been estimated that in 2013, 800,000 people sustained war-related injuries that warranted hospital admission, and approximately 310,000 people died as a consequence of collective violence [2,3]. This however resulted in over 9% of deaths worldwide with over five million deaths annually [4]. It has been projected that armed conflict will become the eighth most important cause of death by 2020 [5].

Africa contributes more to conflict-related deaths than any other region. The United Nations reported 21 neglected crises in the world, of which 17 were in Africa and though, but only a few reports are available on deaths and injuries during armed conflicts in this continent [6]. Studies in eastern Congo (2000 and 2001) have reported millions of deaths among injured individuals, but the specifics are not clear [7].

Elsewhere, the Arab spring uprising is one of the deadliest conflicts in recent history and has been characterized by a human security crisis of extraordinary dimensions. A report published in February 2016 by the Syrian Centre for Policy Research suggests that more than 470,000 people have died in Syria due to the conflict; this number has likely increased but no recent reports seem to exist [8,9]. A report commissioned by the United Nations found that in Yemen, over 30,000 deaths were reported over nearly two years [10]. A recent study carried in Iraq has shown that the fatality rate was 39.1% among individual with intentional injuries. Similar results were also reported in the Syria [11]. Disability subsequent to war-related injury is considered high in countries plagued by years of conflict, which will have a great negative impact on the country’s health perspectives [11]. A cross sectional study carried in Iraq showed that injury incurred more than 4,100 disability-adjusted life-years per 100,000 persons. Most individuals who experience injuries due to armed conflicts usually sustain some kind of temporal or permanent disability; almost 16% of all disabilities reported were due to injury [11,12].

After the NATO leaded war that ousted Colonel Muammar Gaddafi in 2011, Libya entered into a long-standing internal armed conflict. The major armed conflicts between 2011 and 2017 are shown in Table 1. The casualty figures from the conflict and its aftermath are not well documented and debate on conflict-related casualties in Libya continues to this day [13–15]. In 2011, a survey was conducted to determine the mortality, injury and population displacement that occurred during the first year of the conflict. The survey estimated that 21,490 (0.5% of the population) were killed, 19,700 (0.47%) were injured, and 435,000 (10.3%) were displaced. The high rates were reported during May-June, particularly in Tripoli in the western region of the country [16]. Those injured were found to be particularly infected with multi-resistant bacteria such as *Acinetobacter* and *Pseudomonas aeruginosa* [17]. Furthermore, the conflict caused major damage to the health care system. Structural damage was evident in 62 (28.7%) of the health care facilities, 11 (5.1%) were completely destroyed, and 51 (23.6%) suffered partial damage. Primary health care centers accounted for 49 (22.7%) of the damaged facilities, followed by emergency & accident hospitals [18]. However, studies on the consequences of the Libyan armed conflict after 2011 are lacking and there are no adequate data on the burden of the continued conflict on the health care system. Additionally, no studies have been reported on the geographic and spatiotemporal variation of mortality and injury in the country. Hence, studies are needed to assess the burden of the Libyan conflict and to propose appropriate public health action. This study provides an overall assessment of the patterns and trends of mortality, injury and consequent disability related to the Libyan conflict. It also assesses the geographic density of these parameters to show the magnitude and severity of these problems to enable the rational allocation of resources.

**Table 1:** Major operations and armed conflicts in Libya from 2011 to 2017 [46,47,48].

Period	Description
March- November 2011	Operation Odyssey Dawn: NATO military intervention in coordination with Jordan, Qatar, Turkey and United Arab Emirates.
2012–2013	Internal fighting between Libyan militias in most Libyan regions
2014–2015	Operation Libyan Dawn: Western region
2016	Operation Odyssey Lightning: Battle of Serte
2014–2017	Operation Dignity: Eastern region

## Methods

### Setting

This comprehensive national survey was guided by our previously published studies and guidelines [16,17]. Data were collected from all the provinces in the four official regions of Libya. The geographical location, provinces and total population of each region are shown in supplementary S1 Table, S1 Fig.

### Definitions

The individuals included in the study were categorized as dead, injured or disabled according to the following definitions:

**War mortality:** Direct conflict-related deaths caused by weapons and other violent methods strictly used in warfare and reported during the conflict period.

**War-Injury:** Intentional physical harm resulting from fire arms (gun, explosion, air bombardment, *etc*.) that required medical care, whether received or not, and which resulted in loss or reduction in normal activities for a period of time (i.e., study period from 2012 to 2017).

**Intentional disability**: Limitation of normal activities caused by intended deliberate harm, such as gunshots or blasts/explosions reported during the conflict period.

**Exclusion criterion:** Mortality, injuries and disability not related to the war. Detainees, missing persons, deaths consequent to siege conditions (*e*.*g*., starvation) or inability to seek medical care, and injuries and disability caused by road accidents, falls, domestic violence, work-related injuries, and the like. We also excluded any casualties that occurred before or after the study period (2012–2017).

### Study population, data collection and analysis

This study covered individuals aged 15 years or more who have been confirmed as killed or injured as a direct result of the of the conflict between January 1, 2012 and December 31, 2017. Civilian casualties were excluded. Inclusion criteria were death, injury or disability as a direct effect of the armed conflict, and availability of information on date, district, cause of death and demographic characteristics (sex, age and educational level).

Information was collected from the National Death Registry offices in the four Libyan regions, and the Ministry of State for Families of Martyrs, Injuries and Missing Persons [19,20]. Other sources of information were the local authorities in each region. Official government reports and reports of the Libyan Red Crescent were consulted, and accounts were obtained from eyewitnesses and combatants. The information included locations, event types, groups involved, fatalities, injuries, disabilities, demographics, and cause of death or injury. Based on our previously published experience [16,17], an anonymous handwritten questionnaire was developed to collect information from the families of the affected persons; the interviews were conducted by physicians and trained social workers under the supervision of a senior clinical epidemiologist. The purpose was to confirm the data collected from the sources if there was suspicion of inaccuracy or to complete the data if they were incomplete. These data were used to confirm the information on affected people when the available information was incomplete. Moreover, to improve the accuracy of the survey, the final data were discussed with and approved by the local social leaders, revolutionary commanders, and government officials who supported and agreed to assist with the study. The number of deaths and injuries recorded from the different sources is shown in Table 2.

**Table 2:** Data collection sources of the Injured and dead persons during the Libyan armed conflict (2012–2017).

Data source	No. of cases reported	
	Killed	Injured
Regional offices of the National Death Registry	7259	0
Ministry of State for Families of Martyrs, Injuries and Missing Persons [19,20]	5312	17523
Local authorities in each region	721	3087
Official government reports	1251	8901
Reports of the Libyan Red Crescent	830	7901
Eyewitnesses and combatants	371	3120
Families of the affected persons	382	2101

### Geographic mapping

Geospatial analysis was carried out by applying the data of the individuals to the most recently updated electronic map of Libya and its districts showing the municipalities’ borders, on the basis of which distribution maps were constructed. The main spatial descriptive statistics (spatial mean center and spatial standard distance ± 2 standard deviations) were illustrated on these maps. The geographic data from each district involved in the armed conflict was classified according to location, timing and intensity of the Libyan armed conflict from 2012 until 2017. Geo-spatial analysis of mortality and injury were analyzed at the district level as previously described [21].

ArcGIS 10.1 software, Environmental Systems Research Institute Inc., 1999 (ESRI Inc., Redlands, CA, USA) was used to create electronic maps. SPSS18.0 software (IBM Inc., Armonk, NY, USA) was used to analyze the data.

### Statistical analysis

The data were entered in Microsoft Excel and analyzed by SPSS version 12.0 and STAT version 8. Mortality, injury and intentional disability rates per 1000 population per year were calculated on the basis of med-intervals population and we applied long linear regression models in STAT. The rates were consistent among the regions studied over the study period and an additional sensitivity analysis was used to assess the differences in mortality, injury and disability across regions. Mortality and injury rates were compared between the regions for the individual years. As an additional sensitivity analysis, we assessed the effect of differences across regions by extending models to allow the baseline mortality rate to vary by regions and districts. We provide descriptive statistics on cause of death by weapons type. We used R software (version 3.3.2) to calculate proportions and χ^2^ testing.

### Ethical considerations

The approval granted by the Libyan National Ethics Committee (No. LY2012/997/WCS) was credited by the Ethics Board of the Faculty of Medicine, Tripoli. The study was conducted in accordance with the Helsinki Declaration [22]. Written informed consent was obtained from all the participants before the interview and witnessed by the local health officer.

## Results

### Demographic analysis

A total of 16,126 individuals were reported to have been killed and 42,633 injured during the Libyan conflict from 2012 till 2017, as described demographically in Table 3. That translates into a national average mortality rate of 2.7/1000 of the population and an injury rate of 7.1/1000. Males accounted for 81.4% of the conflict-related deaths (CI 95%: 77.3–85.2%) and the difference between the sexes was highly significant (p < 0.001). Mortality was highest among those aged 20–30 years, who accounted for 42.3% of all deaths (CI 95%: 38.1–46.3) (p < 0.001), followed by those aged 31–40 years (26.4% of all deaths; CI 95%: 21.9–30.6; p < 0.001) and then by those aged below 19 years old. The lowest rate was among those aged above 50 years (6.9% of deaths; CI 95%: 3.7–14.2). Those with secondary and primary levels of education had the highest rates of mortality (42.8% of deaths; CI 95%: 37.9–46.2; < 0.001) and 37.9% of deaths (CI 95%: 34.1–41.5; p < 0.001) respectively. Mortality rate was lower among those with a higher level of education (19.3% CI 95%: 14.7–21.9). Furthermore, 55.1% were single (CI 95%: 51.7–59.3; p < 0.001) and 25.6% were married (CI 95%: 21.4–29.2; p = 0.001).

**Table 3:** Demographic and population characteristics of deaths and injuries during the Libyan conflict (2012–2017).

Demographic	Deaths			Injuries		
	Number	% of total (C95%)	p	Number	% of total (C95%)	p
**Sex**						
Male	13,120	81.4 (77.3–85.2)	<0.001	34,380	80.6 (76.2–85.1)	<0.001
Female	3,006	18.6 (13.9–22.4)	0.001	8,253	19.4 (15.1–23.7)	0.001
**Age**						
15–19	2,547	15.8 (11.3–19.5)	0.001	12,435	29.2 (23.9–34.1)	<0.001
20–30	6,819	42.3 (38.1–46.3)	<0.001	6,981	16.4 (12.3–21.1)	0.001
31–40	4,250	26.4 (21.9–30.6)	<0.001	7,137	16.7 (11.2–20.6)	0.001
41–50	1,390	8.6(5.3–12.4)	0.001	14,521	34.1 (30.9–38.3)	<0.001
>51	1,120	6.9 (3.7–14.2)	0.001	1,559	3.7 (2.9–11.3)	0.001
**Educational level**						
Primary	6,112	37.9 (34.1–41.5)	<0.001	19,598	46 (42.1–50.9)	<0.001
Secondary	6,907	42.8 (37.9–46.2)	<0.001	17,719	36.9 (31.9–41.2)	<0.001
Above	3,107	19.3 (14.7–21.9)	0.001	7,319	17.2 (15.8–21.3)	0.001
**Marital status**						
Single	8,890	55.1 (51.7–59.3)	<0.001	20,696	48.5 (44.3–52.2)	<0.001
Married	4,124	25.6 (21.4–29.2)	0.001	12,718	29.8 (24.1–33.2)	0.001
No reported	3,109	19.3 (14.3–24.1)	0.001	9,219	21.6 (18.9–25.7)	0.001
**TOTAL**	**16,126**	**100.0**		**42,633**		

The highest rate of injury was among males (80.6% of all injuries; CI 95%: 76.2–85.1; p < 0.001) particularly among those aged between 41–50 years (34.1%; CI 95%: 30.9–38.3; p < 0.001) followed by those aged below 19 years (29.2% of injured males; CI 95%: 23.9–34.1; p < 0.001). Those above 51 years of age accounted for only 5.1% of injuries CI95%: 2.9–11.3). Most of the injured individuals had primary education (46%) or secondary education (36.9%) (p = 0.001), and only 17.2% had higher education. The injury rate was also higher among single person as it reached (48.5%) though it was only (29.8%) among non-married p> 0.001).

### Trends in mortality and injury

Countrywide, the number of deaths increased steadily from 2012 to 2016, and then decreased slightly. Of all mortalities, 4.9% occurred in 2012, 24.6% in 2016, and 18.6% in 2017.

**Fig 1:**
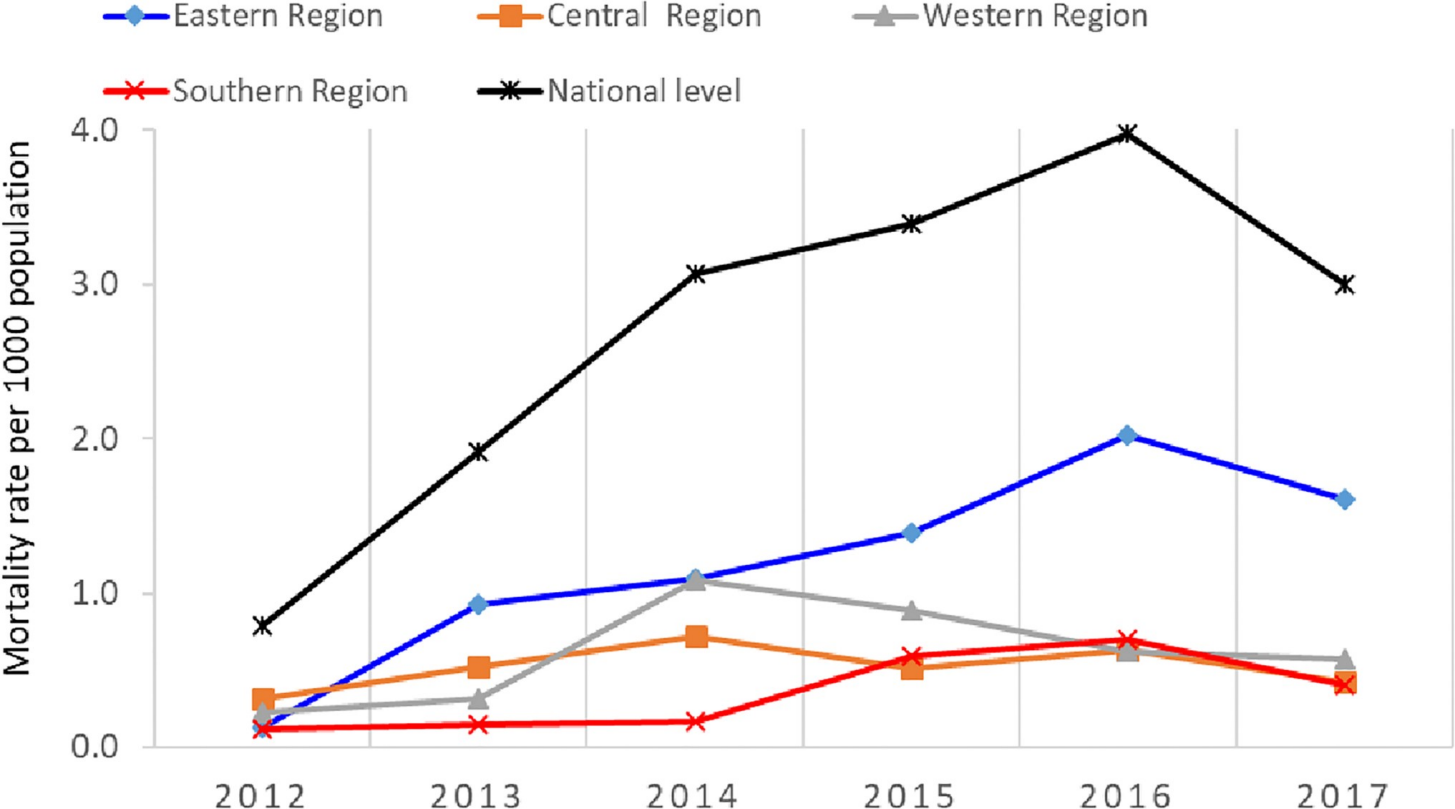
Trends of mortality within the Libyan regions during the armed conflict 2012–2017.

There was considerable variation in mortality between the regions as shown in Fig 1. The largest number of deaths was reported in the eastern region of the country, which accounted for 7164 (44.4%) deaths, followed by the western region with 3713 deaths (23%) and then the central and southern regions, which accounted for 3120 (19.3%) and 2129 deaths (13.2%), respectively.

**Fig 2:**
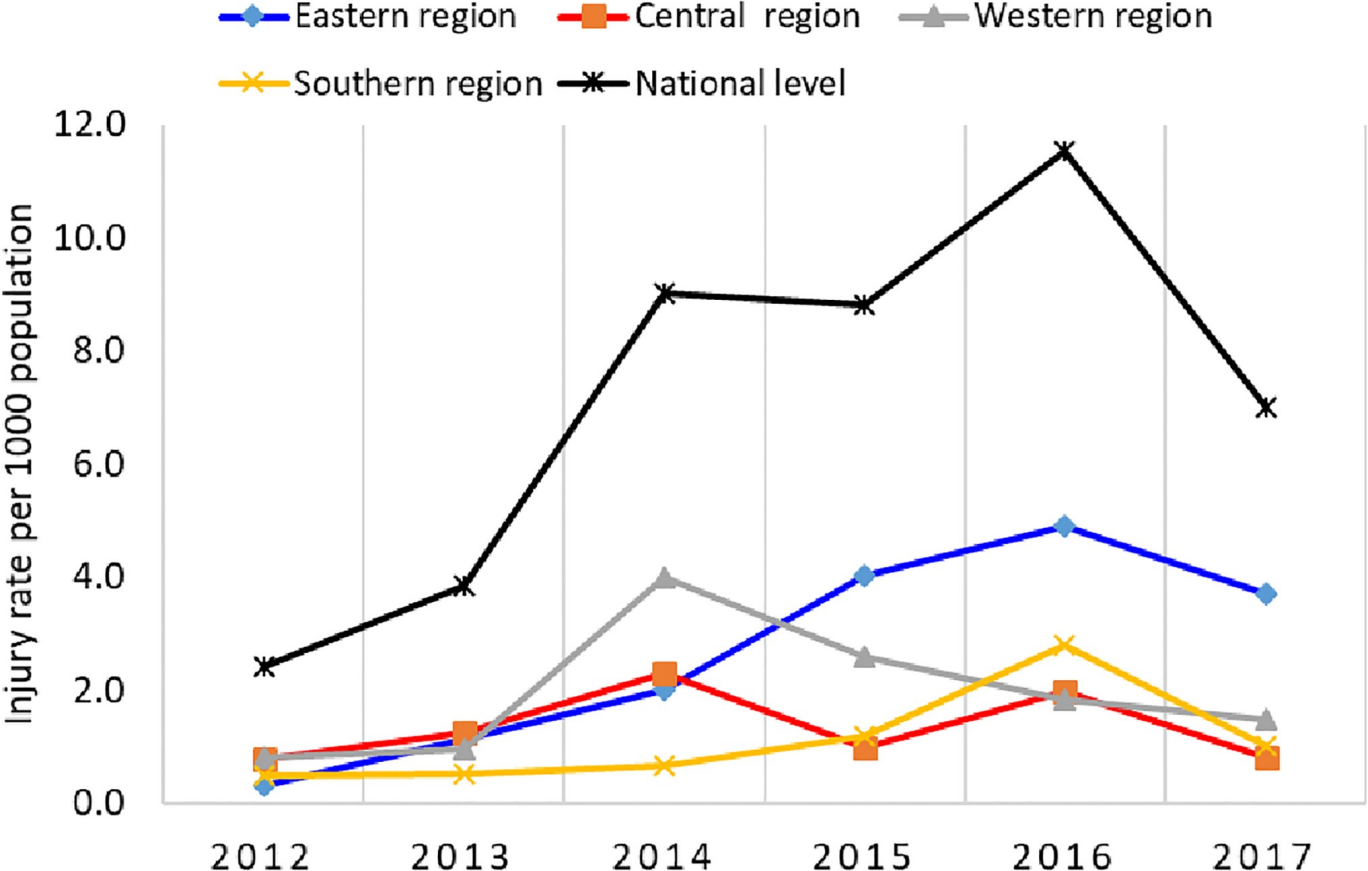
Injury trends within the Libyan regions during the armed conflict 2012–2017.

Fig 2 shows the temporal evolution of the injury rates in each region. The highest incidence of injury was reported within 2014 (21.1%)-2016(27%) which was 2–5 times that of 2012(4.9%) and 2013(11.9%).

### Geographic patterns of mortality and injury

**Fig 3:**
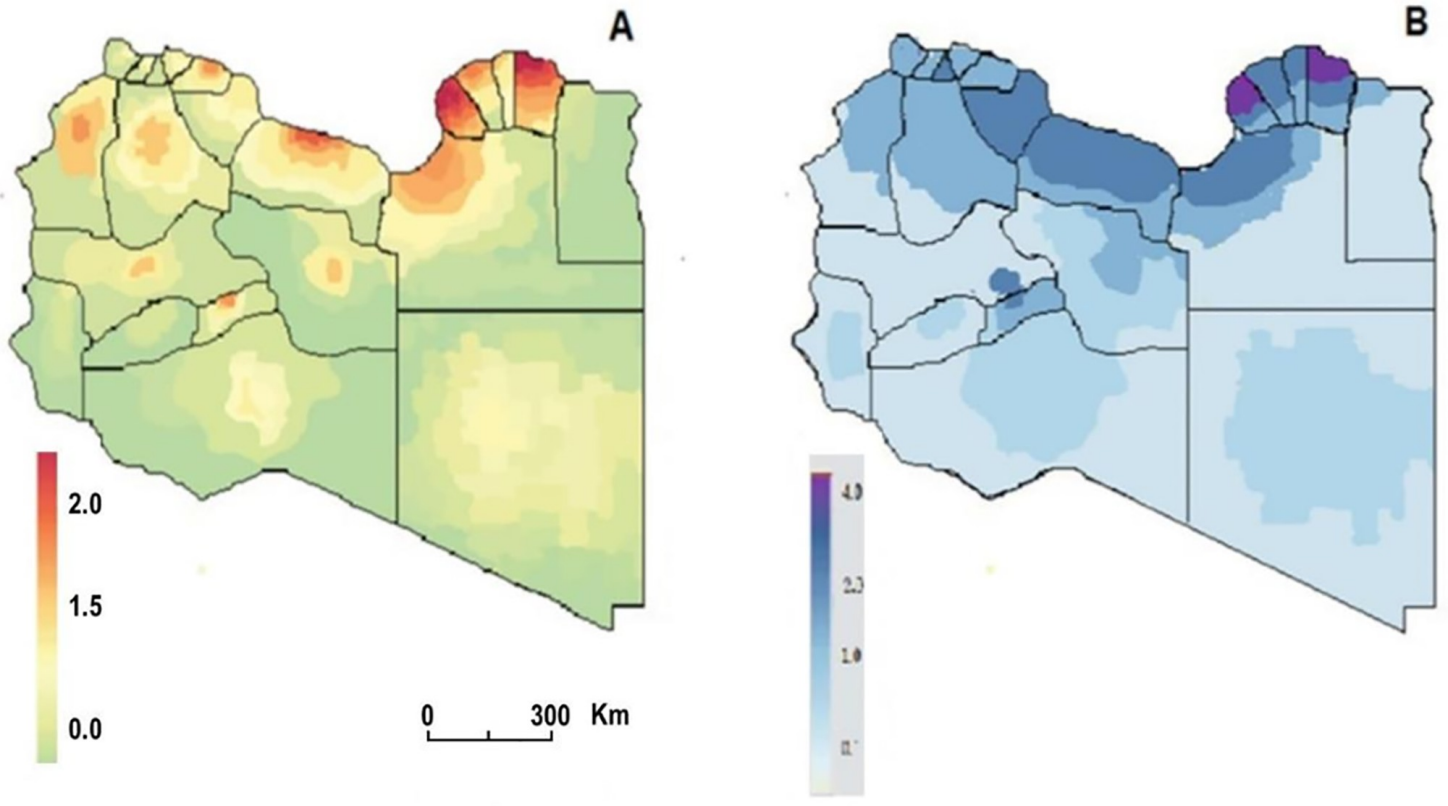
The national geographic patterns of the casualties associated with the Libyan armed conflict. (A) Mortality. (B) Injury. (per 1000 population).

The mortality and injury rates varied substantially between regions and between parts of each region as illustrated in Fig 3. In general, the rate ranged between > 1 death per 1000 to 4 deaths per 1000 (Fig 3A). Districts located in the eastern region experienced the largest increase in death rate during the study period. Benghazi and Derma had the highest rate of mortality (4/1000 population) followed by Jdabia, Mustrata and Sert (2/1000).

The injury rates were about 6/1000 in 10% of the districts, particularly in the eastern and central regions. Though it reached 4/100 in the subcoastal districts which accounted for 20% and 70% of the districts showed a rate of < 1/1000 particularly in Southern and Eastern regions as shown in Fig 3B.

### Consequences of intentional injury

**Fig 4:**
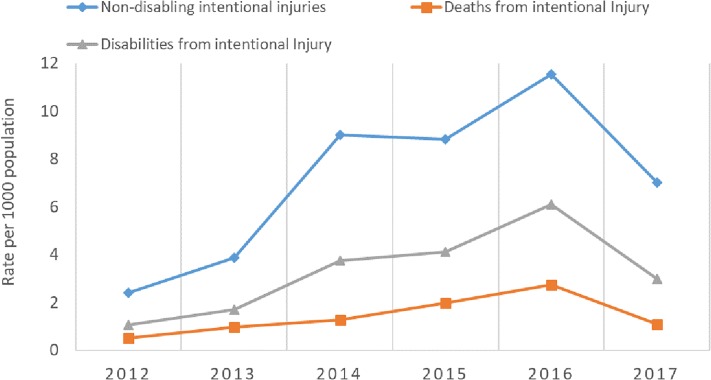
Intentional injury and consequent outcomes from the Libyan armed conflict 2012–2017.

Intentional injury resulted in large numbers of deaths and disabilities during the Libyan conflict as shown in Fig 4. The disability rate increased from a little over 1/1000 population in 2012 to slightly over 6/1000 in 2016. Table 4 shows the relation between the different types of weapons on the one hand and deaths and sustained disability on the other. Most deaths (4721; 55.0%) were caused by gun shots, but sustained disability was associated with blasts and explosions (8710; 61.1%). Survival without disability was higher among those injured by gun shots (10,326; 52.2%) compared with those injured by blasts (3434; 17.4%).

**Table 4:** Types of intentional injury and accompanying deaths and disabilities during the Libyan armed conflict.

Type of Intentional Injury	Number (%)			
	Injuries	Deaths	Disability	Without disability
Gunshot	18,216 (33.1)	4,721 (55.0)	3,169 (22.2)	10,326 (52.2)
Blast /Explosion	14,761 (45.6)	2,617 (30.5)	8,710 (61.1)	3,434 (17.4)
Others	9,656 (21.4)	1,241 (14.5)	2,386 (166.7	6,029 (30.5)
**TOTAL**	**42,633 (100)**	**8,579 (20.1)**	**14,265 (33.5)**	**19,789 (46.4)**

## Discussion

Armed conflicts have heavy, direct and long-lasting impacts, particularly in developing countries, which are ill-equipped to deal with the consequences, and where most wars occur. Armed conflicts are a major cause of mortality, injury and disability, imposing heavy burdens on populations, governments, economies, and health care systems worldwide [23,24]. Documentation of the impact of armed conflict on health is one of the most difficult and most important public health challenges [25]. Injuries and fatalities related directly to the Libyan conflict pose a major challenge to the health care services of the country, in which the already fragile infrastructure has been largely disrupted. Reliable data on the extent, types and geographic distribution of casualties are needed in order to develop rational, targeted health care strategies [26,27].

This study describes the mortality, injury, and disabilities patterns that have resulted from the Libyan armed conflict between 2012 and 2017. The overall mortality and injury rates were 2.7 and 7.1 per 1000 population, respectively. The number of direct conflict-related deaths among men accounted for 81.4% of all conflict deaths. Most of the deaths were among young unmarried people with primary or secondary education levels. Eighty-five percent of them were aged below 40 years, and the highest rate (42.3%) was among those aged 20–30 years. This resembles the pattern in the Iraqi conflict, in which 86% of the deaths were among young males. However, a different pattern was observed in the Syrian conflict, in which the mortality rates among children and women increased as the conflict escalated [28,29]. However, the rates of deaths and injuries among Libyans were higher in 2011 than during the study period of 2012–2017, as it reached over 5/10,000 and 7.2/10,000, respectively.

In contrast to the death rate, the highest rate of injury was among adults over 40 years of age, who accounted for 34.1% of all injuries, followed by young unmarried people below 19 years old (29.2%). Our results resemble those from the Gaza Strip of the Palestinian territory, where males represented 75.5% of the war-related injuries. Almost half (49.5%) of the injured victims were ages 20–39 years, followed by adolescents accounting for 31.4%. More than half of victims were single (53.6%). Similar results were also reported on the Iraqi conflict, in which 51. % of injured people were males aged 20–39 (29.6%) and 33.3% aged 15–19 years [12,30].

Mortality estimates in our study indicate that over 16,000 people were killed during six years. The lowest mortality rate was reported in 2012 and 2013, after which the number of deaths increased, reaching a peak during 2015 and 2016. This varied from one region to another within the country. The largest number of deaths was reported in the eastern region, followed by western and central regions. This finding validates our previous study, which reported the mortalities during the anti-Gaddafi war in 2011.

Logically, the temporal trend in injury rates followed that of the mortality rate, being lowest in 2012–2013 and peaking in 2016. The combined results show that the conflict has been escalating, particularly in the eastern region. The largest number of injured persons was in the eastern region and the lowest was in the southern region. These results are similar to those reported in Kosovo regions during the Balkans conflict but inconsistent with those reported in Iraqi and Palestinian conflicts as the data collected in the later ones was restricted mainly to Gaza strip and the Central area of Iraq particularly Bagdad [31,32].

Intentional or conflict-related injuries are associated with high rates of mortality and disability. A population-based study of war-related deaths in Kosovo in 1999 [33] showed that 64% of the total mortality (8.64/1000 annually) was due to intentional injury. During the 1994 civil war in Afghanistan, war-related injury was the leading cause of death, with 43% (9.4/1000 annually) [34]. There are few reports on injury-related deaths and disability during war or civil conflict in Africa [35]. Our study findings show that intentional injuries resulted in 20.1% death and 33.5% disability. During the Libyan conflict, gunshots were responsible for 33.1% of the injuries and were responsible for 55% the deaths and 22.2% of disability cases. Blasts or explosions were responsible for 45.6% of the injuries and resulted in 30.5% fatality and 61.1% disability. This is similar to reports from Baghdad and the Palestinian territory. Hence then studying the association between weapon types and victim characteristics in armed conflicts could increase understanding of the nature and practices of war and improve individuals’ protection [36,37].

Mapping the extent of damages, population displacement and tense of killing are rarely reported [38]. The uniqueness of this study, it applied for the first time, a geographic mapping approach for an army conflict area, which is rarely reported before. We efficiently visualized the patterns of mortality and injury during the Libyan conflict. The analysis showed that certain districts have been plagued by the conflict for a longer period. Spatial variation is noticeable within these regions, particularly Derna, Ajdabia and Benghazi, followed by Sert, Musrata, Tripoli and Jufra. The heaviest clusters of conflict-related mortality were in Benghazi and Derna in the east of the country and Sert in the central region. This could be related to socioeconomic factors and the ideological beliefs of the fighting groups in these areas. However, further studies are needed to understand such speculations. Our study indicates that there are important opportunities to more closely examine districts and regions with notably high mortality and injury rates. Such information is an important input for developing an effective public health response policy to combat the consequences of the conflict [39,40]. [38,39]

## Limitations

Though this study attempted to be comprehensive and was based on documented registry data, it might not fully represent the actual situation. First, many cases might not have been reported because of the vast area of the country and the lack of security [41]. Second, as the study lasted for six years, the registration cannot be easily sustained particularly for minor and short cases of injury and disability. Moreover, the study did not highlight the severity of injuries, degrees and duration of disabilities, and whether injured victims had recovered. This may have affected the accuracy of the number of deaths and injuries reported. Fourth, locations were frequently estimated, which could have led to imprecision. Fifth, civilian deaths and injuries are not well illustrated because the use of heavy weapons blocked roads and turned certain urban areas into no-go zones [42]. “We are pursuing this line of research by investigating civilian casualties, and particularly children.”

## Conclusions

The Libyan war has inflicted a grave toll on the country’s population and public health system. The overall fatality and injury rates increased from 2012 to 2017, with a peak in 2016. Young men comprised more than 80% of the dead and injured. Analyzing mortality and injury burdens on even narrower geographic scales is important for guiding public health responses and allowing more effective targeting.

## Future directions

The Libyan armed conflict has and will continue to impose heavy burdens on the demography and quality of life of Libyan society. The country faces serious and unprecedented challenges. No single policy can be addressed in a complex and relapsing conflict environment such as Libya [43,44]. Hence, the country should be alerted to deal with such ongoing consequences particularly within the health care settings. The Libyan health care system has to be re-organized to deal with these consequences by adopting short-term and long-term strategies [45]. These may include the following:

- Establishment of a national electronic registry system for persons injured or disabled as a consequence of the conflict.
- Data collection and follow up should be carried as the conflict consequences goes beyond parameters estimates and more likely that Injury and disability outcomes to be reflected and felt by the society for many years ahead.
- The pervasive impact of Libyan armed conflict is long-term and not confined to combat-related deaths. Injured and disabled victims are largely young males, making rehabilitation and integration programs a necessity.
- Further studies are needed to assess the advancement in the complications and the new perspectives of the Libyan armed conflict.

## Supporting information

S1 Table Libyan regions, districts, administrative boundaries and population density. (RAR)

S1 Fig Map of Libya showing the geographical locations and administrative boundaries of the regions and districts covered in the study. (RAR)

## Abbreviations

NATO: North Atlantic Treaty Organization
